# Quantification of blood loss for the diagnosis of postpartum hemorrhage: a systematic review and meta-analysis

**DOI:** 10.1590/0034-7167-2023-0070

**Published:** 2023-12-04

**Authors:** Mariana Torreglosa Ruiz, Nayara Freitas Azevedo, Cynthya Viana de Resende, Wellington Francisco Rodrigues, Joilson Meneguci, Divanice Contim, Monika Wernet, Carlo José Freire de Oliveira

**Affiliations:** I Universidade Federal do Triângulo Mineiro. Uberaba, Minas Gerais, Brazil; II Universidade Federal do Triângulo Mineiro, Hospital de Clínicas. Uberaba, Minas Gerais, Brazil; III Universidade Federal de São Carlos. São Carlos, São Paulo, Brazil

**Keywords:** Quantification of Blood Loss, Postpartum Hemorrhage, Postpartum Period, Review, Meta-Analysis, Cuantificación de la Pérdida de Sangre, Hemorragia Postparto, Periodo Posparto, Revisión, Metaanálisis, Quantificação de Perda Sanguínea, Hemorragia Pós-Parto, Período Pós-Parto, Revisão, Metanálise

## Abstract

**Objective:**

to compare the effectiveness of different diagnostic methods to estimate postpartum blood volume loss.

**Methods:**

a systematic review of effectiveness according to PRISMA and JBI Protocol. Searches in PubMed/MEDLINE, LILACS, Scopus, Embase, Web of Science and CINAHL, with descriptor “Postpartum Hemorrhage” associated with keyword “Quantification of Blood Loss”. Tabulated extracted data, presented in metasynthesis and meta-analysis was applied to quantitative data. To assess risk of bias, JBI Appraisal Tools were applied.

**Results:**

fourteen studies were included, published between 2006 and 2021. Quantification of loss by any method was superior to visual estimation and is highly recommended, however the studies’ high heterogeneity did not allow estimating this association.

**Conclusion:**

the studies’ high heterogeneity, with a probable margin of error given the uncontrolled factors, indicates the need for further studies, however quantification proved to be effective in relation to visual estimate. PROSPERO registration CRD 42021234486.

## RESUMO

**Objetivo:**

comparar a efetividade de diferentes métodos diagnósticos para estimar a perda volêmica sanguínea pós-parto.

**Métodos:**

revisão sistemática de efetividade, de acordo com protocolo PRISMA e JBI. Buscas nas bases PubMed/MEDLINE, LILACS, Scopus, Embase, *Web of Science* e CINAHL, com o descritor “*Postpartum Hemorrhage*” associado à palavra-chave “*Quantification of Blood Loss*”. Dados extraídos tabulados, apresentados em metassíntese, e aplicou-se metanálise para dados quantitativos. Para avaliar o risco de viés, aplicou-se o *JBI Appraisal Tools*.

**Resultados:**

incluídos 14 estudos, publicados entre 2006 e 2021. A quantificação da perda por qualquer método apresentou superioridade em relação à estimativa visual, sendo altamente recomendada, porém a alta heterogeneidade dos estudos não permitiu estimar essa associação.

**Conclusão:**

a alta heterogeneidade dos estudos, com provável margem de erro dado aos fatores não controlados, indica a necessidade de realização de novos estudos, contudo a quantificação se mostrou efetiva em relação à estimativa visual. Registro PROSPERO CRD 42021234486.

## RESUMEN

**Objetivo:**

comparar la efectividad de diferentes métodos diagnósticos para estimar la pérdida de volumen sanguíneo posparto.

**Métodos:**

revisión sistemática de efectividad según PRISMA y Protocolo JBI. Búsquedas en bases de datos: PubMed/MEDLINE, LILACS, Scopus, Embase, Web of Science y CINAHL, con el descriptor “Postpartum Hemorrhage” asociado a la palabra clave “Quantification of Blood Loss”. Los datos extraídos tabulados fueron presentados en metasíntesis y metanálisis se aplicaron a los datos cuantitativos. Para evaluar el riesgo de sesgo, se aplicaron las herramientas de evaluación de JBI.

**Resultados:**

se incluyeron 14 estudios, publicados entre 2006 y 2021. La cuantificación de pérdida por cualquier método fue superior a la estimación visual y es muy recomendable, sin embargo, la alta heterogeneidad de los estudios no permitió estimar esta asociación.

**Conclusión:**

la alta heterogeneidad de los estudios, con probable margen de error dado el descontrol de los factores, indica la necesidad de más estudios, sin embargo la cuantificación demostró ser efectiva en relación a la estimación visual. Registro PRÓSPERO CRD 42021234486.

## INTRODUCTION

Postpartum hemorrhage (PPH) is defined as blood loss greater than 500 ml after vaginal childbirth or greater than 1,000 ml in cesarean section in the first 24 hours, or any blood loss after childbirth capable of causing hemodynamic instability and/or requiring blood transfusion for its control. It is an avoidable, complex and multicausal problem, since the clinical response to postpartum blood loss is variable and can be influenced by several factors, including the correct diagnosis. Thus, accurate diagnostic methods are effective in identifying losses, such as avoiding, correcting and/or minimizing the poor prognosis in cases of hemorrhages that have already started^(1-5)^.

Although preventable and treatable, PPH is responsible for about 27% of obstetric-related deaths^(1-5)^. It is estimated that, for every ten childbirths, there is one case of PPH and one death for every 190 childbirths^(2,4-6)^. This number becomes even more alarming when it is verified that more than 800 women in the world die every day, due to complications related to pregnancy and/or childbirth^(1-5)^, with the frequent contribution of PPH conditions to these numbers. It is noteworthy that, in the Brazilian scenario, from 1996 to 2020, PPH was the cause of 17.3% (5,056 deaths) of maternal deaths, second only to complications arising from hypertensive syndromes, whose percentage reached 57.9% of causes in the period^(7)^.

However, a diversity of diagnostic methods described in the literature and used in worldwide assistance are identified, the most frequently cited being: visual estimation of bleeding; weighing compresses and surgical drapes used in childbirth care, known as gravimetry; use of graduated and calibrated collecting devices; diagnosis through clinical parameters and clinical estimation through the shock index; and comparison of hemoglobin (Hb) and/or hematocrit (Ht) concentration 24 hours postpartum and sample collected at the end of pregnancy. Recently, colorimetry has been investigated and used, which is an artificial intelligence system using a 32 GB Apple iPad Pro Wi-Fi device connected via Bluetooth to an application (Triton^®^) via a wireless network^(2,4,8-10)^. It should be noted that accurate methods that allow timely diagnosis, at a lower cost and that allow its realization in the most diverse scenarios, are decisive for the identification and treatment of case as well as for women’s prognosis and quality of life.

Given the alarming rates of PPH in Brazil and in the world, one of the main causes of maternal death, the diversity of diagnostic strategies for its identification, given that an accurate diagnosis constitutes a major challenge in the management of this obstetric emergency and that rapid treatment contributes to a better prognosis and reduction of morbidity and mortality, this study is justified.

## OBJECTIVE

To compare the effectiveness of different diagnostic methods to estimate postpartum blood volume loss.

## METHODS

### Study design

This is a systematic review of effectiveness. According to the JBI, systematic reviews of effectiveness are able to identify whether an intervention, used appropriately, achieves the expected effects^(11)^.

To carry out the review, steps were taken based on JBI recommendations^(11)^. The study was registered in the International Prospective Register of Systematic Reviews (PROSPERO) database - protocol CRD 42021234486, structured according to the Preferred Reporting Items for Systematic Reviews and Meta-Analyses Protocol (PRISMA)^(12)^ and JBI recommendations^(13)^.

To formulate the review question, the PICO framework was used, where P (population): postpartum women; I (intervention): diagnostic methods to quantify postpartum blood volume loss; C (comparator): visual estimation of blood loss; O (outcomes): postpartum blood loss. These elements made up the review question: what is the effectiveness of different diagnostic methods to quantify postpartum blood volume loss in postpartum women compared to visual estimation of blood loss?

### Data collection

Searches were carried out in September 2022 independently by two reviewers, one with a doctor’s degree (MTR) and the other with a master’s degree (NFA), using controlled descriptors from Medical Subject Headings, CINAHL Headings, Embase Emtree and Health Sciences Descriptors, with the term “Postpartum hemorrhage” associated with the keyword “Quantification of blood loss”. The descriptor postpartum hemorrhage was added to the search, since quantification of blood loss is not a controlled descriptor. Thus, it was decided to add the descriptor for sensitive search. It is noteworthy that the search strategy was validated by a librarian with experience and qualification in sensitive searches.

Searches were performed in the following databases: US National Library of Medicine National Institutes of Health (PubMed); Web of Science; Excerpta Medica DataBASE (Embase); SciVerse Scopus; Cumulative Index to Nursing and Allied Health Literature (CINAHL); and Latin American and Caribbean Literature on Health Sciences (LILACS). The choice of databases was due to the number of primary articles in health indexed in these databases. The objective with the diversity of databases is to contemplate the world production on the theme.

The following strategy was used for the MEDLINE/PubMed search: (postpartum hemorrhage [MeSH Terms] OR postpartum hemorrhage OR postpartum bleeding or hemorrhage, Postpartum Immediate or Hemorrhage, Immediate Postpartum or Postpartum Hemorrhage, Immediate or Delayed Postpartum Hemorrhage or Hemorrhage, Delayed Postpartum or Postpartum Hemorrhage, Delayed) AND (Quantification of blood loss). This strategy was used as a standard for searching the other databases, being slightly modified, based on the specific criteria of each database, as shown in Chart 1.

**Chart 1:** Search strategy in the consulted databases, 2022

Database	Search strategy in March 2022
PubMed/ MEDLINE	(postpartum hemorrhage [MeSH Terms] OR postpartum hemorrhage OR postpartum bleeding OR hemorrhage, Postpartum Immediate OR Hemorrhage, Immediate Postpartum OR Postpartum Hemorrhage, Immediate OR Delayed Postpartum Hemorrhage OR Hemorrhage, Delayed Postpartum OR Postpartum Hemorrhage, Delayed) AND Quantification of blood loss
CINAHL	(MH “Postpartum Hemorrhage/PC”) OR TI ((“postpartum hemorrhage” OR “postpartum bleeding” OR pph OR “postpartum haemorrhage”) OR AB ((“postpartum hemorrhage” OR “postpartum bleeding” OR pph OR “postpartum haemorrhage”) AND (quantification of blood loss)
Embase	(postpartum hemorrhage) AND (quantification of blood loss)
LILACS	(*Hemorragia Pós-Parto* OR Postpartum Hemorrhage OR *Hemorragia Posparto*) AND (Labor Stage, Third OR *Terceira Fase do Trabalho de Parto* OR *Tercer Periodo del Trabajo de Parto*) (*prevenção & controle* or prevention & control or *prevención & control*) AND (quantification of blood loss)
Web of Science	(postpartum hemorrhage) AND (quantification of blood loss or gravimetric measurement of blood)
Scopus	(postpartum hemorrhage OR postpartum bleeding OR hemorrhage, Postpartum Immediate OR Hemorrhage, Immediate Postpartum or Postpartum Hemorrhage, Immediate OR Delayed Postpartum Hemorrhage OR Hemorrhage, Delayed Postpartum OR Postpartum Hemorrhage, Delayed) AND (quantification of blood loss)

**Chart 2:** Characteristics of the studies included in the review (N = 14), 2022

Author(s) and reference	Year of publication	Country	Objectives	Number of participants	Design	Intervention and comparator	Outcomes	Main findings	Risk of bias^*^
Al Kadri HM, Al Anazi BK & Tamim HM^(29)^	2011	Saudi Arabia	Compare the accuracy of visually estimated postpartum blood loss with gravimetry.	150 women who had vaginal childbirths	Cohort study Included: women who had vaginal childbirths Excluded: postpartum women who received blood transfusion within 24 hours after childbirth and those who had antepartum hemorrhage	Intervention: gravimetry Control: visual estimation (VE) Reference: drop in Hb level	There was a difference between the means of 304.1 ml (gravimetry) and 213-214 ml (VE). There were no differences between the VE performed by a doctor or a nurse. VE led to 30% in the estimation error, when compared to drop in Hb.	Gravimetry showed a statistically significant difference.	8/11 (each participant was their own control, and confounders and strategies were not anticipated in these cases)
Ambardekar S, Shochet T, Bracken H, Coyaji K, Winikoff B^(23)^	2014	India	Compare the results of quantification of blood loss with gravimetry techniques using a calibrated field.	900 women who had vaginal childbirths (the sample size calculated to test a 50 ml difference between techniques, with 80% power and 5% significance, was 900 women, and 450 were allocated to each study arm)	Randomized clinical trial Inclusion criteria: over 18 years old who had vaginal childbirth as an outcome; excluded women undergoing cesarean section Allocated into 02 groups (intervention and comparator) Losses measured from cord clamping to 1 hour after childbirth or until the bleeding stops. Hb dosage was performed 24 hours after	Intervention: BRASSS-V drape^®^ (graduated and calibrated field) Comparator: gravimetry Reference: drop in Hb level 24 hours after childbirth	The mean estimate was 253.9 ± 218.2 (20 - 1600 ml) for the calibrated field group and 195.3 ± 201.8 (20 - 2000 ml) for the gravimetry group.	There were important differences in quantification. The calibrated and graduated field showed greater accuracy and should be considered in care protocols. As limitations, the authors cited the failure to record the start and end time of collection, losses during weighing/measurement that were not measured, fluid replacement was not calculated, and that the study’s testing was only for vaginal childbirths.	11/13 (there is no information regarding blinding of participants and evaluators)
Blosser C, Smith A, Poole AT^(17)^	2021	United States	Analyze differences between VE and calibrated field quantification of postpartum blood loss.	5,445 childbirths, regardless of the type of childbirth Groups: 828 cesarean sections in the field group and 848 VE, 1,877 vaginal in the field group and 1,883 VE	Cohort study Exclusion criteria: gestational age less than 20 gestational weeks Analyzed the need for blood transfusion based on drop in Ht	Intervention: calibrated and graduated field Comparator: VE Reference: drop in Ht level	There was a difference between the means in the groups, with 2.1% of losses greater than 1,000 ml being detected in VE and 6.5%, when quantified by the field. Quantification proved to be the best predictor for assessing the need for blood transfusion for all types of childbirth.	Quantification of blood loss was the most sensitive test to detect clinically significant losses.	8/11 (each participant was their own control, and confounders and strategies were not anticipated in these cases)
Doctorvaladan SV, *et al.* ^(18)^	2017	United States	Compare the accuracy of VE and gravimetry techniques with colorimetry to determine post-cesarean blood loss.	50 women undergoing cesarean section	Observational study Compared VE, gravimetry and colorimetry from an automated device	Intervention: colorimetry Comparators: VE and gravimetry Reference: drop in Hb level	Different means were obtained when compared with the techniques: 928 ml for VE; 822 ml for gravimetry; and 572 for colorimetry.	Colorimetry proved to be the best predictor for measuring blood loss, using drop in Hb as a reference, showing greater accuracy.	8/11 (each participant was their own control, and confounders and strategies were not anticipated in these cases)
Hire MG, Lange E, Vaidyanathan M, Armour K L & Toledo P^(19)^	2020	United States	Determine whether blood loss colorimetry can reduce interventions and diagnoses of PPH in cesarean childbirths compared with VE.	42 cesarean sections	Observational study Cases in which visual loss greater than 1,000 ml was estimated were selected.	Intervention: colorimetry Comparator: VE Reference: Hb dosage	The mean visually estimated was 1,275 ml (1,100-1,510 ml). The mean obtained by colorimetry was 948 ml (700-1,267 ml). 57% of visually estimated cases were not classified as PPH by the device.	There was an overestimation of loss, when assessed by VE, and PPH protocols could have been installed unnecessarily.	8/11 (each participant was their own control, and confounders and strategies were not anticipated in these cases)
Katz *et al.^(^* ^20)^	2020	United States	Assess the impact of quantification by calibrated field in the estimation of postpartum blood loss compared with colorimetry.	7,781 childbirths 2,568 vaginal childbirths by calibrated field 2,541 by colorimetry 1,243 cesarean sections per calibrated field 1,266 by colorimetry	Observational study Compared to quantification by weighing with calibrated and graduated field and colorimetry	Intervention: quantification with calibrated field Comparator: colorimetry	Quantification in vaginal childbirths (300 ml per field and 258 ml by colorimetry). For cesarean sections, 800 ml per field and 702 ml per colorimetry.	Statistically significant differences were found in the estimation of cases of hemorrhage, being recommended in this study quantification by calibrated field.	8/11 (each participant was their own control, and confounders and strategies were not anticipated in these cases)
Kearney L, Kynn M, Reed R, Davenport L, Young J & Schafer K.^(26)^	2018	Australia	Assess the effectiveness of gravimetry in estimating PPH.	522 vaginal childbirths	Cohort study Exclusion criteria: undergoing cesarean section; multiple pregnancies	Intervention: gravimetry Comparator: VE	Gravimetry made it possible to identify 70% of cases of PPH with a positive correlation (r: 0.88). VE estimated 78% of these cases.	VE was more accurate compared to gravimetry in this study, raising questions about the use of routine gravimetry after uncomplicated childbirths.	8/11 (each participant was their own control, and confounders and strategies were not anticipated in these cases)
Khadilkar SS, Sood A & Ahire P^(24)^	2016	India	Assess the differences between VE and gravimetry and the effectiveness of simulated training for quantification.	100 vaginal childbirths (36 primiparous and 64 multiparous); 50 cesarean sections (31 primiparous and 17 multiparous); and 50 simulated scenarios VE and gravimetry were compared and, afterwards, the identification of PPH in simulated scenarios	Prospective observational study The following participated as loss evaluators: 20 nurses; eight anesthetists; 20 residents in obstetrics; and six professors of obstetrics	Intervention: training with simulated scenarios and use of VE and gravimetry Comparator: prior knowledge from VE and gravimetry	There was an improvement in the accuracy of losses greater than 500 ml after training. The error rate was 42% to 58% before training, and reduced to 12% to 30% after training. The highest percentage of error was associated with VE, in which the loss was underestimated.	VE was not reliable, and the training had an impact on improving accuracy.	8/11 (each participant was their own control, and confounders and strategies were not anticipated in these cases)
Lertbunnaphong T, Lapthanapat N, Leetheeragul J, Hakularb, P & Ownon A^(27)^	2016	Thailand	Compare the effectiveness of VE with gravimetry to determine the volume of postpartum blood loss.	286 women who had vaginal childbirth at term, where patients were their own controls.	Prospective observational study Inclusion criteria: over 18 years old, full-term vaginal childbirth, low-risk pregnancy	Intervention: gravimetry Comparator: VE	There was a significant difference in postpartum blood loss between IV (178.6 ml) and gravimetry (259 ml). There were losses of less than 100 ml - an underestimation of 27.6% of VE. 65.4% of cases of PPH were misdiagnosed by VE (underestimation).	There was an underestimation of losses when using VE. It is suggested to replace the technique with gravimetry.	8/11 (each participant was their own control, and confounders and strategies were not anticipated in these cases)
Lilley G, *et al.^(^* ^28)^	2015	England	Validate the accuracy of quantification by gravimetry through a simulated scenario.	18 simulated scenarios with the participation of 25 obstetricians, 21 anesthetists, 36 nurse midwives and 18 actors, nine anesthesia assistants and eight midwifery students in training Observational: 348 childbirths (gravimetry and Hb drop) - 205 gravimetry <1,500 ml and 143 > 1,500 ml	Methodological study (scenario validation) and observational Only cases classified as PPH were observed.	Intervention: gravimetry Comparator: VE	In scenario validation, the measurement error rate was 34.7% in VE and 4% in gravimetry. The gravimetry used in the observational study showed a positive correlation for the detection of cases of PPH (r = 0.80), and 40% of the women had losses greater than 1,500 ml.	Gravimetry proved to be more accurate for identifying large blood losses. The authors point out that the technique does not require more resources than a baby scale and basic math skills, and can be taught and used routinely in all maternity services.	8/11 (each participant was their own control, and confounders and strategies were not anticipated in these cases)
Lumbreras-Marquez MI, *et al.* ^(8)^	2020	United States	Compare PPH detection in vaginal and cesarean childbirths after standardization of quantification of blood loss by colorimetry.	2,468 childbirths: 967 pre-device and 645 post-device vaginal childbirths; 456 pre-device and 418 post-device cesarean sections	Observational study Inclusion criteria: singleton pregnancies Preand post-implementation results of the device were analyzed	Intervention: colorimetry Comparator: gravimetry Reference: preand postpartum Ht level	The mean obtained by the device was 237± 522 ml. By gravimetry, the mean found was 600 ± 556 ml. The difference in means was 349 ml. Device use increased the diagnosis of PPH by 2.49 times.	The device increased the chance of detecting PPH in vaginal childbirths, however there were no statistically significant differences in the preand post-implementation period of device use.	9/11 (confounders and strategies were not anticipated in these cases)
Patel A, *et al.* ^(25)^	2006	India	Compare VE with the calibrated and graduated field estimate and difference between the calibrated field estimate and colorimetry.	123 vaginal childbirths	Randomized and controlled clinical trial Inclusion criteria: vaginal childbirth 61 patients allocated to VE 62 for the graduated field estimate 10 first from each group, colorimetry being performed	Intervention: calibrated and graduated field Comparator: VE Reference: colorimetry	The mean VE loss was 203 ml (50-950 ml). The mean obtained by the assessment per field was 304 ml (50-975 ml) in the field group, with a mean difference of 101 ml. The mean blood loss with colorimetry was 188 ml (93-286 ml), while the mean blood loss using the drape method was 239 ml (100-350 ml), with a mean difference of 51 ml. A correlation of 0.928 was obtained, indicating accuracy of quantification by field.	The estimation of blood loss by field quantification proved to be more accurate than VE.	11/13 (it was not possible to blind participants and researchers)
Rubens-tein AF, *et al.* ^(21)^	2021	United States	Compare quantification of blood loss from colorimetry with VE.	274 vaginal childbirths, where patients were their own controls	Observational study	Intervention: colorimetry Comparator: VE	The mean obtained by colorimetry was 339 ml (217-515) and significantly higher than the VE, whose mean was 300 ml (200-350). Loss >500 ml was detected in 73 (26.6%) patients compared to 14 (5.1%) patients using VE. PPH (losses greater than 1,000 ml) was recorded in 11 patients (4.0%), while only one patient was diagnosed with PPH by VE.	Quantification by colorimetry proved to be more accurate for the diagnosis of PPH and excessive losses. The study points to the need for objective quantification and suggests further clinical studies using the resource to verify its effectiveness.	8/11 (each participant was their own control, and confounders and strategies were not anticipated in these cases)
Toledo P, McCarthy RJ, Hewlett BJ, Fitzgerald PC, Wong CA ^(22)^	2007	United States	Test the hypothesis that calibrated and graduated fields are more effective than VE.	106 professionals from the obstetric team of a university hospital - obstetric anesthetists and nurses - after undergoing training using simulation - there was no difference between groups	Quasi-experimental study Eight stations were simulated with different amounts of bleeding - 300 to 2000 ml (with and without urine, with and without amniotic fluid). Calibrated and graduated fields and uncalibrated (visual) fields were used.	Intervention: calibrated and graduated fields Comparator: VE without calibrated and graduated field	There was an improvement in VE to assess greater amounts of post-training blood. There was no difference in quantification with calibrated and uncalibrated fields. Calibrated fields showed an error rate of less than 15% and provided better measurement accuracy.	Calibrated and graduated fields showed better accuracy, being a low-cost resource.	8/9 (before-and-after study, no control group)

### Selection criteria

Primary studies, with experimental, quasi-experimental or observational designs, that addressed the effectiveness of diagnostic methods to estimate postpartum blood volume loss, without delimitation of language and time, were included. Study design is justified because, according to JBI recommendations, the evidence that assessed the effectiveness of interventions comprises the three main categories of studies: experimental; quasi-experimental; and observational^(11)^.

Duplicate articles in the databases, studies with secondary data (reviews), opinion articles, consensus, guidelines, research protocols, letters to the editor, articles with designs different from those eligible and articles that did not answer the review question were excluded.

The PRISMA^(12)^ methodology was adopted to systematize the process of inclusion of studies and illustrated in a flowchart. Duplicate articles, with study design inappropriate to the question and those that did not answer the review question were excluded. Full texts were selected in a paired and independent way, and those that met the eligibility criteria were selected for the study.

Study selection was carried out independently by two researchers and disagreements were resolved by consensus. It should be noted that there was no need to include a third researcher for conflict resolution, although it was initially foreseen in the project.

The searches resulted in 134 studies.

### Data analysis and treatment

In the first step, duplicates were removed (n = 28) and 59 articles were excluded after reading the titles and abstracts (53 did not answer the review question and six were reviews). After the first selection, 47 articles were read in full and at this stage 35 were excluded, 29 for not portraying the review question and six due to study design (protocols, case studies, recommendations). Reading the studies in full made it possible to manually identify two studies that were cited in the included articles (references of the analyzed reference). Thus, the final sample consisted of the analysis of 14 studies. The sequence of sources analyzed in the databases was PubMed^®^, Embase, CINAHL, LILACS, Scopus and Web of Science™.

The methodological quality assessment tools of JBI (JBI Appraisal Tools)^(13)^ were used to identify the methodological quality and risk of bias of the studies included individually, using the versions suitable for experimental, quasi-experimental and observational studies. This step was also performed by two researchers (MTR and NFA), independently.

Two independent researchers (MTR and NFA) extracted detailed and standardized information by JBI^(13)^, such as details about publication and study, authors and reference, year, producing country, objectives, number of participants, design, interventions performed and comparators, measurement of outcomes, main findings related to the review question and risk of bias (methodological quality assessment). The extracted data were tabulated and presented descriptively.

The quantitative data of the studies were stored in Excel Microsoft^®^ spreadsheets, and for data analysis and visual display, the programs RStudio 4.2.1 and “Prism” from Graphpad (version 8.0) were used.

The General Package for Meta-Analysis “meta” version 4.9-5 was used for the assessment between variables with the application of the “metamean” command, and the Relative Risk (RR) with the respective Confidence Intervals (CI) was used as a measure of association. The forest plot was used for data assessment and representation. Study heterogeneity was assessed using the I^2^ statistic from Cochran’s Q test and the number J of analyzed studies. The random effect model was applied to all associations^(14-16)^.

## RESULTS

A total of 14 studies were included in the analysis, the first publication dates from 2006 and the last from 2021, all in English. Seven studies (50%) were produced in the United States^(8,17-22)^ and three in India^(23-25)^ (21.4%). Australia^(26)^, Thailand^(27)^, England^(28)^, Saudi Arabia^(29)^ were represented by one study from each country (7.1%, respectively).

There were different methods researched in the studies according to the producing country. In American studies, there was a higher prevalence of studies comparing visual estimation, however, the national production was responsible for the entire world production in which the colorimetry method was used. In turn, Indian studies more frequently compared simple gravimetry with quantification by calibrated field. Saudi Arabia and England were producers of studies in which quantification through drop in Hb was compared. Visual estimation was the most adopted technique (38.6%), and the comparison of pre and postpartum Hb levels, the least used (4.5%).

By design, eight (57.1%) were observational/cross-sectional, three (21.4%) were prospective cohorts, two (14.2%) were randomized controlled trials, and one study (7.1%) was quasi-experimental. One study used a methodological approach and was then assessed by observational design (7.1%).

The application of tools for assessing the methodological quality and risk of bias from JBI Tools made it possible to identify a low risk of bias in all included studies. However, even with good methodological quality, some of the items were not met in the studies, according to their design. In observational studies, the most neglected items were controls (participants were their own controls), and confounders and strategies were not anticipated in these cases. In clinical trials, however, it was not possible to blind participants and researchers, since strategies/methods are visually different. It should be noted that, due to the theme under study in the cases presented, it is not possible to meet the items presented above, which did not compromise study results or quality.

Adding up all births in which postpartum blood loss was assessed, there was a total observation of 20,763 childbirths, 5,341 measurements of bleeding in cesarean sections and 14,378 vaginal childbirths. In a study, 1,044 observations were made without description of the type of childbirth, whose data were analyzed together, without differentiation.

It is noteworthy that from one childbirth it was possible to compare two or more techniques and, in most studies, postpartum women were their own control. Furthermore, there was an analysis of 18 simulated scenarios in one study^(28)^ and eight simulated stations with different amounts of bleeding, allowing comparison of two methods^(22)^, which resulted in the sum of 26 simulated scenarios.

Figure 2 assesses the differences between the observations made (childbirths and simulated scenarios). The scenarios were simulated with greater losses, simulating PPH conditions and presented homogeneity. However, differences were found when comparing the two types of childbirth, with greater losses in cesarean sections, but, due to the studies’ high heterogeneity, it was not possible to determine the influence of the type of childbirth on the bleeding means in the analyzed studies.

**Figure 1:**
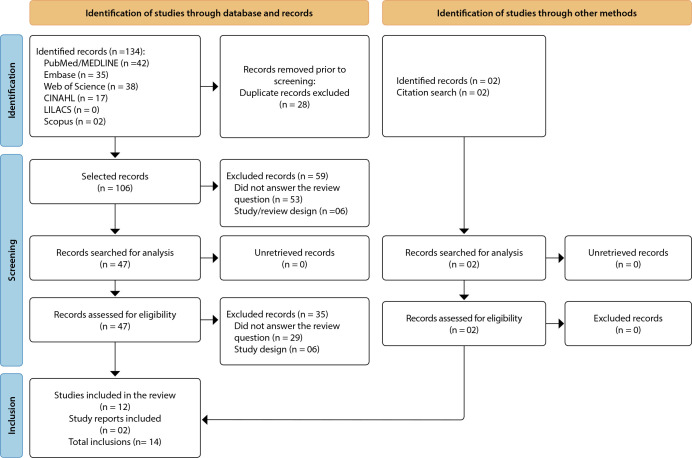
PRISMA 2020 flowchart for systematic reviews that include searches in databases, registries and other sources

**Figure 2:**
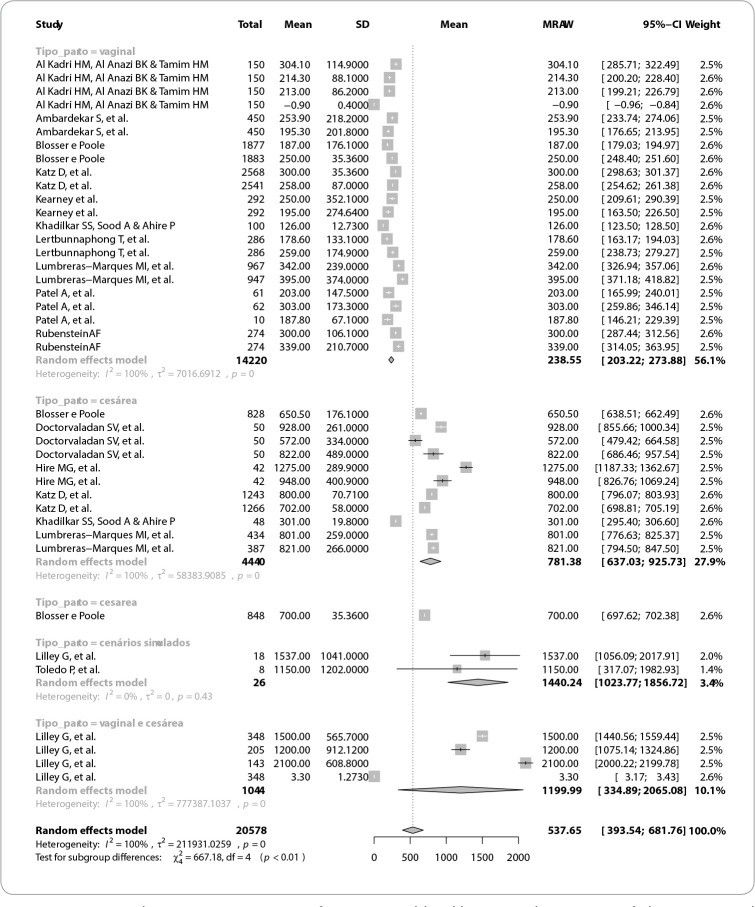
Meta-analysis comparing means of postpartum blood loss according to type of observation and forest plot of the mean differences found according to the method used

**Figure 3:**
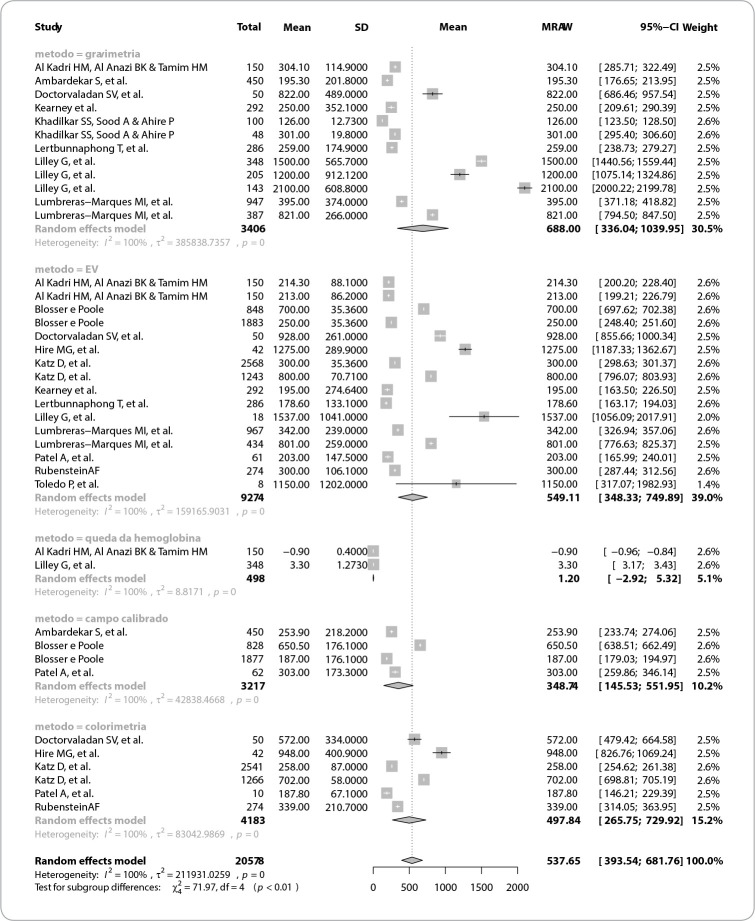
Meta-analysis on different methods of quantifying postpartum blood loss and forest plot of the mean differences found, according to the method used, 2022

The studies’ and techniques’ high heterogeneity indicates that there is probably a margin of error given possible uncontrolled factors predicted in the studies, which should be reported in new primary studies. Figure 3 shows the meta-analysis on different methods of quantifying postpartum blood loss and forest plot of the mean differences found, according to the method used.

Thus, the high heterogeneity of the studies included in the analysis did not allow identifying the most effective method for quantifying postpartum bleeding. However, it is noteworthy that any form of identification was superior to visual estimation and that the simulated scenarios promoted an improvement in the care provided.

## DISCUSSION

Accurately estimating blood loss in the third period of childbirth is a daily challenge for obstetric care worldwide, with the strong limitation being the fact that most studies that quantify blood loss refer mainly to general surgical patients, not having focus on obstetric population^(30)^.

Of the analyzed studies, there was a predominance of observations of vaginal childbirths (79.3%). According to the literature, vaginal childbirth is the childbirth route with the greatest challenges for measuring blood loss, since it is not possible to control variables related to secretions present during labor and the immediate postpartum period, such as amniotic fluid and urine, which may overestimate or underestimate the estimates made in the different methods^(31-32)^. Moreover, it is noteworthy that in the countries producing the analyzed studies, high rates of normal childbirth predominate, which may have favored the increase of this type of childbirth in the analysis^(31-32)^.

In this meta-analysis, there was greater blood loss in cesarean sections when compared to vaginal childbirths, confirming that, as in the definition of PPH, blood loss is different according to the type of childbirth^(2,4)^. This situation of greater loss in the surgical act was also contemplated in the simulated scenarios presented in the study.

VE was described more frequently, as in the literature, which points to it as the most used method in obstetric care for this purpose^(33-35)^. It is noteworthy that its accuracy depends on the control of several variables, such as the expertise and experience of the professionals who are assessing it, being considered subjective and difficult to reproduce. Although it presents zero costs, especially in scenarios with a greater amount of bleeding, VE is usually flawed, with overestimation being described in large losses and underestimation in smaller but continuous losses^(35-39)^, which can waste unnecessary resources, such as transfusions, delay diagnosis and treatment, and may compromise postpartum women’s health.

Studies that assessed the use of gravimetry, through simple weighing, showed great heterogeneity, similar to what was found in other studies^(40-41)^. The heterogeneity is justified by the lack of control of the variables, their confounders or even the criteria established in the study methods. Studies point out as possible confounders and limitations of the technique the presence of other liquids (amniotic, diuresis, sweating) that can overestimate the loss^(40-41)^, absence of well-defined protocols^(41)^ and the fact that, in case of failures in the communication between the obstetric team and anesthesia, it is not possible to perform a strict water balance, especially regarding serum therapy^(42)^. Furthermore, the possibility of human error in accounting for losses is highlighted^(41)^. Thus, the need for primary studies on the technique is reinforced.

The need for a accuracy scale, periodically calibrated, is also described as a difficulty for carrying out gravimetry. The authors describe the need to weigh towels, sheets, swabs, cotton swabs, pads, gauze, compresses, clots and subtract these materials from the dry weight and suggest that they be weighed dry and wet on the same scale to avoid bias. Furthermore, there is the factor that many of these materials can absorb the liquid part of the blood, causing it to evaporate and translate into a smaller quantity^(42)^.

When analyzing the methods comparing VE versus the quantitative methods, the studies pointed out that the quantitative methods are more inclined to detection with greater accuracy in cases of PPH^(19,21,25,27,29,31,36)^.

Quantification is recommended for the diagnosis of PPH in all types of childbirth, regardless, applied to lowand high-risk postpartum women. It is able to reduce maternal morbidity; provides care in a timely manner; provides objective measurement, which impacts on PPH recognition and treatment (reduces delays and supports decision-making); reduces the administration of uterotonics and unnecessary transfusions; consists of a tool to rescue women in PPH; requires greater staff awareness, but does not increase the workload; timely mobilizes additional resources (intensive care bed and transfusions); it contributes to the early and conscious use of uterotonics when necessary and, consequently, presents better results when compared to the VE of bleeding^(36)^; and it is also a low-cost resource, as it only requires calibrated scales and trained professionals.

It is strongly recommended to create protocols and bundles associated with training the team to adopt the technique, which is a recommendation from the American College of Obstetricians and Gynecologists. It is also pointed out that institutions that adopted quantification reduced costs with unnecessary PPH treatments, unnecessary transfusions, and enhanced the diagnosis of PPH cases^(31,36)^.

Although the impact of the use of calibrated and graduated fields (collection bags attached to the parturient woman) was not observed in the present review, a review study of quantification of blood loss in vaginal childbirths showed greater accuracy than gravimetry^(9)^. Since it is a low-cost resource, its use should also be considered in clinical practice, associated with quantification.

Clinical assessment methods, including monitoring changes in vital signs (heart rate and blood pressure) and assessing the shock index as well, are strongly recommended resources in practice for the diagnosis of PPH^(35)^. However, it is noteworthy that the physiological response to hemorrhage is a crucial and determining factor for the early recognition of a high-risk situation, but that the methodologies must be associated for an early and more assertive identification^(33-35,42-43)^.

A resource with high accuracy is the measurement of Hb and/or Ht before and after childbirth. The results of the review showed a lower frequency of studies that compared techniques with hematimetric dosage, however drop in Hb or Ht was a reference parameter for studies that compare different quantification techniques. In this regard, a systematic review study that investigated methods used to measure postpartum blood loss indicated that, although accurate, quantification by laboratory dosage is rarely used and can be difficult in certain scenarios depending on the context of the institution and even the country’s reality, due to the costs with the technique^(9)^. It should be noted that a study found a correlation between a 10% drop in the Ht level on admission and Hb levels below 9 mg/dl and that in these cases, women had lipothymia and mucosal discoloration^(44)^, reinforcing the benefit of their job when possible.

Colorimetry is the method with the most recent description in the literature. The analysis pointed out its use only in American studies. It is one of the most accurate methods for quantification; however, it is the one that involves greater costs and complexity. Its measurement takes place by reading the image of sponges, compresses or fields with blood, through artificial intelligence through applications^(31)^. Results point out as an advantage the lower bias in relation to other methods and the possibility of measuring in real time^(34)^. From the evidence described, it is suggested to reflect on the possibility of use in institutions.

As pointed out in a review on quantification of loss in the case of vaginal childbirths^(9)^, the evidence presented was not sufficient to support one method over another, due to the high heterogeneity, due to controlled and uncontrolled factors, with a probable margin of error due to uncontrolled factors. Thus, new primary studies are suggested, preferably randomized and controlled clinical trials with methodological rigor.

The results showed greater homogeneity in studies involving simulated scenarios and improvements in quantification after training professionals. There is an exponential increase in the use of realistic simulation in preparing professionals for emergencies, and more specifically in obstetric emergencies, including PPH^(45)^. Studies prove that simulation is capable of promoting cognitive and behavioral education and provides meaningful learning for the participants involved in the scenario, with superior results than other teaching strategies and methodologies^(46)^. It constitutes an important strategy to increase the clinical experience of both students and professional health teams, in addition to promoting improvements in care, ensuring patient safety, maximizing learning and limiting the frequency and impact of possible adverse events in care^(47-48)^, being highly recommended in cases of PPH.

The results showed that the more the assistance team is trained in the methods of quantifying volume loss in the postpartum period, the less divergences and the more reliable the quantifications. These data are corroborated by results found in other studies in which the use of simulation in cases of PPH was investigated^(36,46,49-50)^.

When looking at the characteristics of the countries that produced the studies included in the analysis, 71.4% (n=10) were carried out in developed countries and used strategies with greater technological complexity for comparison with VE, mainly. It is necessary to reflect that in the world, one woman dies for every 190 births and, although there are large differences between the countries of the same continent, the risk of dying from maternal causes is greater in developing and underdeveloped countries. In Asian countries, the chance of dying from PPH is 1:280 births; in Africa, 1:39; in countries belonging to Oceania, 1:170; and in European countries, 1:4,300^(5)^. It is noteworthy that it is in these countries (developing and underdeveloped) that financial resources are scarcer and effective and more accessible prevention and early identification methods need to be implemented with greater urgency^(4)^.

### Contributions to nursing and health

Considering the evidence presented, the need for a trained team with a diagnostic method compatible with the local care reality is evident for a more reliable identification and safe management of PPH, reducing maternal mortality rates from this cause. The need to carry out more homogeneous studies with controlled variables in terms of methodology and challenges according to the type of childbirth is highlighted, preferably investigations with randomized and controlled clinical trials.

### Study limitations

As a limitation, we can mention that, due to the studies’ high heterogeneity, evidence was not sufficient to indicate the most effective method for quantifying postpartum blood loss. However, at the same time that it constitutes a limiting factor, it becomes an opportunity for developing new studies on the subject.

## CONCLUSIONS

The high heterogeneity of eligible studies, with a probable margin of error due to uncontrolled factors, did not allow us to identify the most effective method for quantifying postpartum blood loss. However, quantification of blood loss by any method was superior to VE and is highly recommended, regardless of the technique.

The use of simulated scenarios as a resource for training the team resulted in improvements in quantification of bleeding and in the recognition of PPH cases, being strongly recommended as well as the adoption of updated protocols and bundles.

It is noteworthy that it is up to managers to know their care reality as well as the methods for quantification, results and costs involved, in order to establish the best cost-benefit ratio.

There is a need for new primary studies, mainly randomized and controlled clinical trials on the different methodologies for estimating blood loss, given their relevance to obstetric care.
